# Fabrication, characterization and high photocatalytic activity of Ag–ZnO heterojunctions under UV-visible light

**DOI:** 10.1039/d1ra05060e

**Published:** 2021-08-10

**Authors:** Xiaodong Zhu, Juan Wang, Daixiong Yang, Jiawei Liu, Lili He, Mao Tang, Wei Feng, Xiaoqiang Wu

**Affiliations:** College of Mechanical Engineering, Chengdu University Chengdu 610106 China fengwei233@126.com wuxiaoqiang@cdu.edu.cn

## Abstract

Pure ZnO and Ag–ZnO nanocomposites were fabricated *via* a sol–gel route, and the obtained photocatalysts were characterized by XRD, SEM, TEM, BET, XPS, PL and DRS. The results showed that Ag^0^ nanoparticles deposit on the ZnO surface and Ag modification has negligible impact on the crystal structure, surface hydroxyl group content and surface area of ZnO. However, the recombination of photogenerated electrons and holes was suppressed effectively by Ag loading. The photocatalytic activity was investigated by evaluating the degradation of MB under xenon lamp irradiation as the UV-visible light source, and the results show that the photocatalytic activity of ZnO significantly improved after Ag modification. Ag–ZnO photocatalysts exhibit higher photocatalytic activity than commercial photocatalyst P25. The degradation degree of MB for 1%Ag–ZnO was 97.1% after 15 min. ˙O_2_^−^ radicals are the main active species responsible for the photodegradation process, and Ag–ZnO heterojunctions generate more ˙O_2_^−^ radicals, which is the primary reason for the improved photocatalytic performance.

## Abstract 2

Ag–ZnO heterojunction promotes the separation of photogenerated pairs and thus exhibits high catalytic activity under UV-visible light.

## Graphical abstract

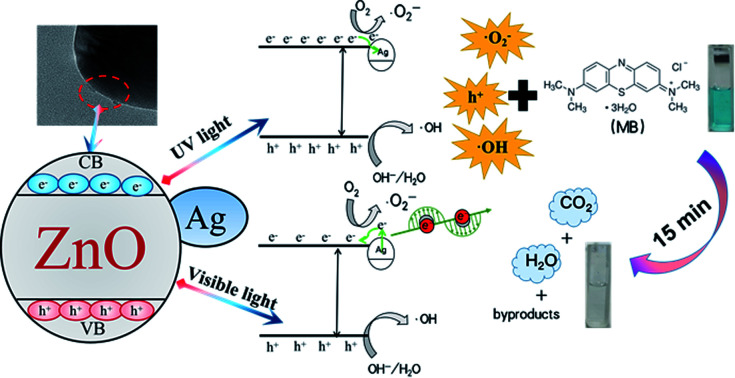

## 1 Introduction

Metal oxide semiconductors have been regarded as a kind of promising materials to deal with pollution due to their photocatalytic activity. Among them, ZnO, an important semiconductor material, has attracted an extensive range of research attention because of its low cost, excellent electrochemical stability and high electron mobility.^1–4^ However, there are also some defects in ZnO. It is usually active in the presence of UV light and the photoexcited electrons and holes recombine easily, which results in a decrease of its photocatalytic property.^5–10^ Therefore, advancing the photocatalytic activity of ZnO has become an active research topic at present. ZnO modified by noble metal loading^11–14^ and ion doping^15–18^ has attracted considerable attention and has been widely studied. The effect of ion doping on the photocatalytic performance of ZnO is still controversial. Liu *et al.*^15^ found that Fe doping can modify the ZnO nanofiber morphology and reduce the optical bandgap to improve its photocatalytic activity. However, ion doping does not necessarily improve the photocatalytic performance of ZnO, and it has been reported that Fe–doped ZnO and Cu-doped ZnO show less photocatalytic activity than pure ZnO.^17,18^

For noble metal loading, the photocatalytic activity of ZnO can always be enhanced by loading with Au,^19^ Pt^20^ and Ag.^21^ Unexpectedly, there are controversies involved in the explanation of noble metal deposition for improving the photocatalytic performance of ZnO. Muñoz-Fernandez *et al.*^20^ synthesized Ag–ZnO nanocomposites by a solvothermal method and are convinced that the enhancement of photocatalytic activity lies in the increased surface area and less recombination rate of photogenerated pairs compared to pure ZnO. The Ag–ZnO hybrid with high photocatalytic activity was prepared by Dong *et al.* and they showed that the high activity of Ag–ZnO was due to a high degree of crystallinity and a higher ratio of surface hydroxyl oxygen.^22^

In the sol–gel method, it is easy to add some impurity elements evenly and quantitatively to achieve uniform doping at the molecular level, thus obtaining photocatalysts with high photocatalytic activity. On the other hand, although several studies on Ag–ZnO composites have been reported, the photocatalytic activity of each study is quite different and there are numerous controversies about the mechanism of Ag modification to improve the photocatalytic activity of ZnO. Therefore, in the present study, different concentrations of Ag modified ZnO were prepared by the sol–gel method, focusing on the effects of Ag modification on the crystal structure, surface morphology, elemental composition and valence state, optical properties and photocatalytic activity of ZnO. Ag–ZnO heterojunction photocatalytic materials with activity higher than P25 were obtained, and the mechanism of Ag modification on improving the photocatalytic activity was analyzed.

## 2 Experimental

### 2.1 Fabrication of pure and Ag–ZnO nanocomposites

Pure and Ag–ZnO nanocomposites were fabricated as follows: diethanolamine (6 mL), Zn(CH_3_COO)_2_·H_2_O (13.2 g) and certain amounts of AgNO_3_ were dissolved in a 160 mL ethanol/water system (the volume ratio is 5 : 3). A ZnO sol could be obtained after stirring at 70 °C about 120 min and aging for 2 h. The resulting mixture solution was dried at 90 °C and then heated at 550 °C for 2 h. Finally, 1%, 3% and 5% (Ag/Zn molar ratio) Ag-loaded ZnO nanocomposites can be obtained after full grinding. The 1% Ag-loaded ZnO is labeled as 1%Ag–ZnO, same as 3%Ag–ZnO and 5%Ag–ZnO. Pure ZnO was prepared for comparison by the same procedure without adding AgNO_3_.

### 2.2 Characterization

X-ray diffraction (XRD) was used to examine the crystal structure of samples using an X-ray diffractometer (DX-2700). The specific surface areas were determined by nitrogen adsorption–desorption isotherms using the Brunauer–Emmett–Teller (BET) theory. X-ray photoelectron spectra (XPS) test by a spectrometer (XSAM800) was employed to examine the chemical state. A field-emission scanning electron microscope (SEM, FEI-Inspect F50) and a transmission electron microscope (TEM and HRTEM, FEI-Tecnai G2 F20) were used to observe the surface morphology. The recombination rate of photoinduced electrons and holes was investigated by photoluminescence (PL) spectra using a luminescence spectrometer (F-4600). UV-vis absorption spectra were recorded on a spectrophotometer (UV-3600).

### 2.3 Photocatalytic test

The photocatalytic property of pure and Ag–ZnO was assessed by examining the photocatalytic degradation of MB. The experiments were conducted as follows: MB solution (100 mL, 10 mg L^−1^) and 100 mg sample were added into a beaker. To establish the adsorption–desorption equilibrium between MB molecules and photocatalysts, the obtained mixture was stirred for 30 min in dark. A 250 W xenon lamp was employed as the UV-visible light source. About 5 mL sample was removed at a given time interval and centrifuged to completely remove the suspended photocatalyst particles. Then, the MB absorbance at 664 nm was measured *via* a UV-Visible spectrophotometer. The degradation degree was calculated by the equation: 
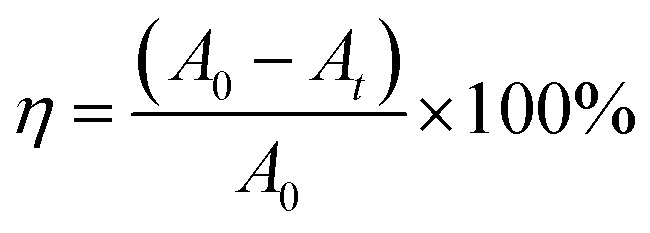
, where *A*_0_ and *A*_*t*_ are the initial absorbance and absorbance at time “*t*”, respectively.

## 3 Results and discussion

### 3.1 Crystal structure

**Fig. 1:**
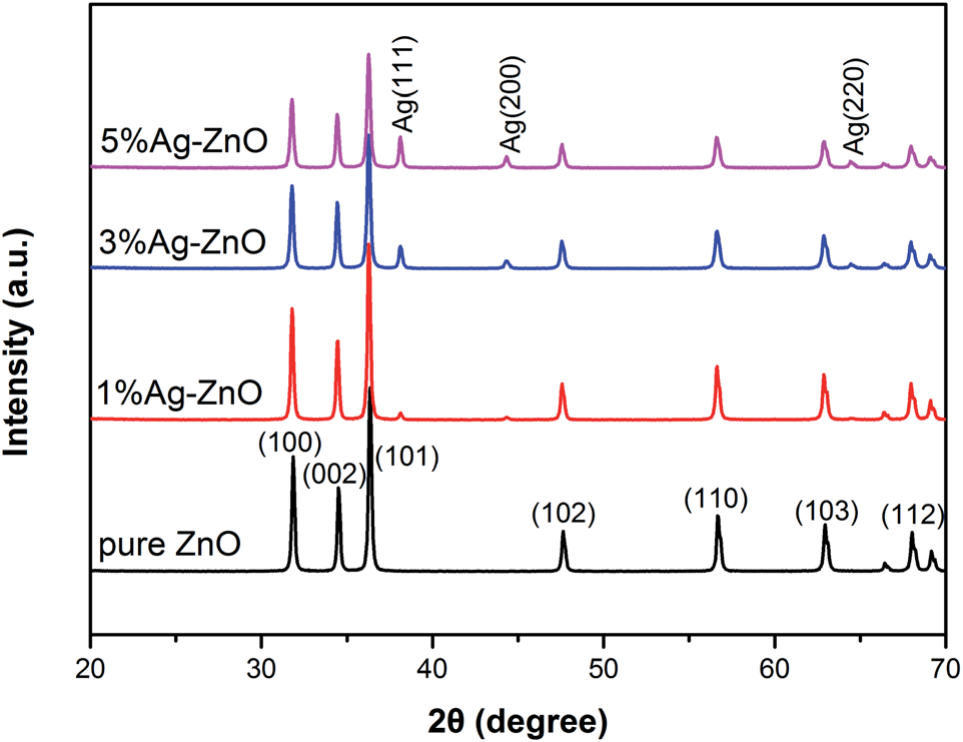
XRD patterns of pure ZnO and Ag–ZnO nanocomposites.

Fig. 1 shows the XRD patterns of pure ZnO and Ag–ZnO nanocomposites. All peaks are strong and sharp, which indicates that both pure ZnO and Ag–ZnO are highly crystalline. The pure ZnO pattern displays diffraction peaks at 31.8°, 34.4°, 36.3°, 47.6°, 56.6°, 62.9° and 68.0°, which are indexed to the crystal planes of (100), (002), (101), (102), (110), (103) and (112) of hexagonal wurtzite structures, respectively.^1,13^ The shape and position of peaks in Ag–ZnO patterns are similar to those of pure ZnO, indicating that Ag–ZnO nanocomposites are also hexagonal wurtzite structures. Moreover, the peaks at 38.1°, 44.3°, 64.5° in Ag–ZnO patterns are assigned to the (111), (200) and (220) planes of the cubic Ag phase,^20,23^ implying the existence of metallic Ag in Ag–ZnO nanocomposites. The intensity of these peaks gradually increases with the increase in the Ag addition content, indicating that more metallic Ag forms in the matrix. On the other hand, as the Ag concentration increases, the peak intensity of ZnO decreases, suggesting that the crystallinity and grain size decrease in Ag–ZnO samples.^7,19^ The grain sizes (*D*) of pure ZnO, 1%, 3% and 5%Ag–ZnO nanocomposites were 38.8 nm, 38.6 nm, 35.5 nm and 35.2 nm, respectively, which were calculated by Scherrer's formula *D* = 0.89*λ*/*β* cos *θ*.^5,17^ The grain size is slightly reduced by Ag loading, which can be attributed to the fact that Ag particles exist on the ZnO surface, blocking the migration and diffusion of ZnO.^9^

### 3.2 Morphology and BET surface area

**Fig. 2:**
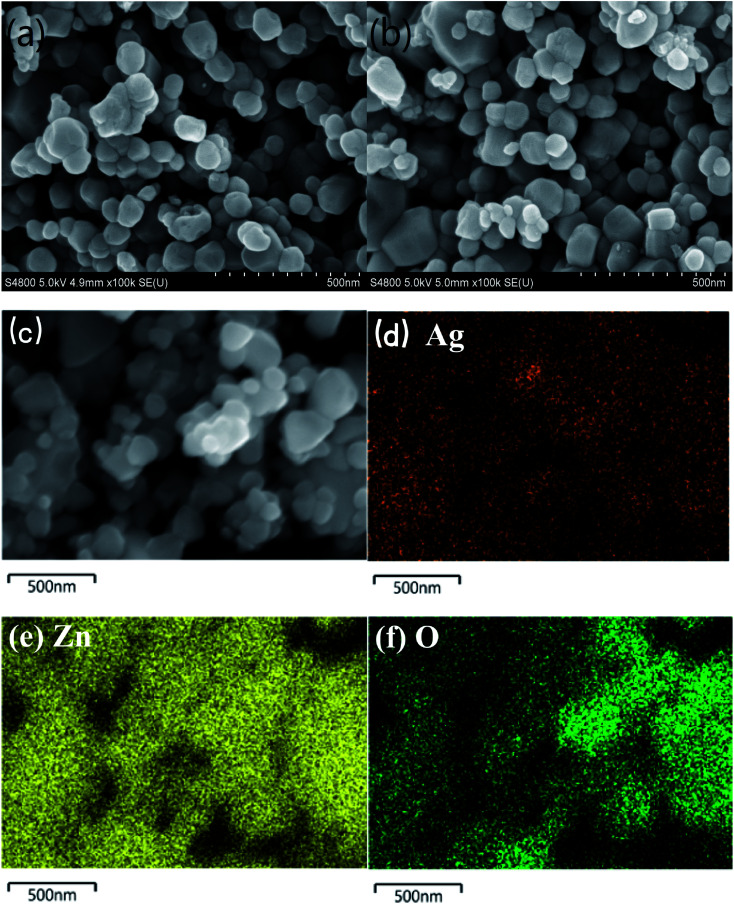
SEM images of pure ZnO (a), SEM images (b) and EDS mapping (c–f) of 1%Ag–ZnO.

Fig. 2 shows SEM images of pure ZnO (a) and 1%Ag–ZnO (b) nanocomposites. It was observed that the particles in pure ZnO represent spherical-like shape with diameter in the range of 40 nm to 100 nm. The particles in 1%Ag–ZnO show a similar spherical-like morphology. The EDS mapping (Fig. 2(c)–(f)) of 1%Ag–ZnO reveals that Zn, O and Ag elements exist and were uniformly dispersed in the matrix.

**Fig. 3:**
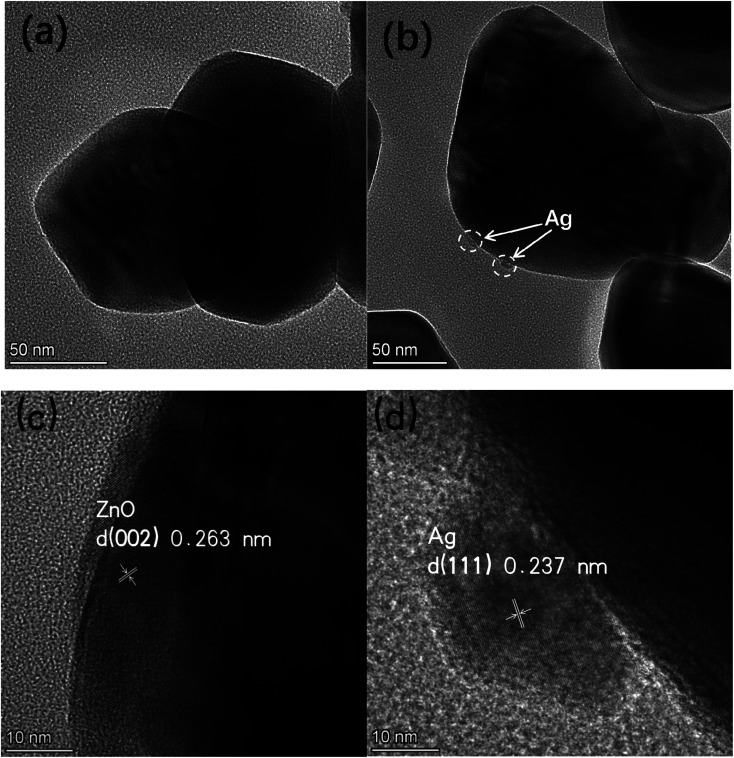
TEM images of pure ZnO (a), 1%Ag–ZnO (b), HRTEM images of pure ZnO (c) and 1%Ag–ZnO (d).

The TEM images of pure ZnO (a) and 1%Ag ZnO (b) are displayed in Fig. 3. It can be seen that the particle sizes in pure ZnO were 40–100 nm approximately, which is in line with the SEM image. In the 1%Ag–ZnO sample, Ag nanoparticles with the size of 5–10 nm can be observed to be deposited on the surface of ZnO. Fig. 3(c) and (d) display the HRTEM images of pure ZnO and 1%Ag–ZnO, respectively. The lattice distance in Fig. 3(c) is 0.263 nm, which corresponds to the (002) plane of hexagonal ZnO.^22,24^ It is observed in Fig. 3(d) that a particle deposited on the matrix and the marked lattice distance is 0.237 nm, ascribing to the (111) plane of metallic silver, which suggests that Ag particles is absorbed on the surface of ZnO.^25,26^ The TEM results are in line with the XRD and XPS results, proving that the Ag element exists as metallic silver.

It is well known that the specific surface area is a key factor to study the photocatalytic property of ZnO. However, the impact of Ag loading on the specific surface area of ZnO is disputed. Numerous studies reveal that the specific surface area of ZnO increases after the addition of Ag.^27–29^ On the contrary, Ag-modified ZnO exhibits less specific surface area compared to the pure ZnO in Liang's^30^ study. Moreover, Zhu *et al.*^31^ found that the specific surface area is almost unchanged after the Ag loading. The different results of specific surface area may be attributed to numerous syntheses methods and processes. In the present study, the BET surface areas of pure ZnO and 1%Ag–ZnO obtained were 5.7 m^2^ g^−1^ and 5.8 m^2^ g^−1^, respectively. SEM and TEM images show that the morphology of Ag–ZnO does not change significantly compared to that of pure ZnO. Accordingly, the specific surface area of ZnO was not influenced by Ag loading, which is in agreement with literature.^31^

### 3.3 Elements and chemical state

**Fig. 4:**
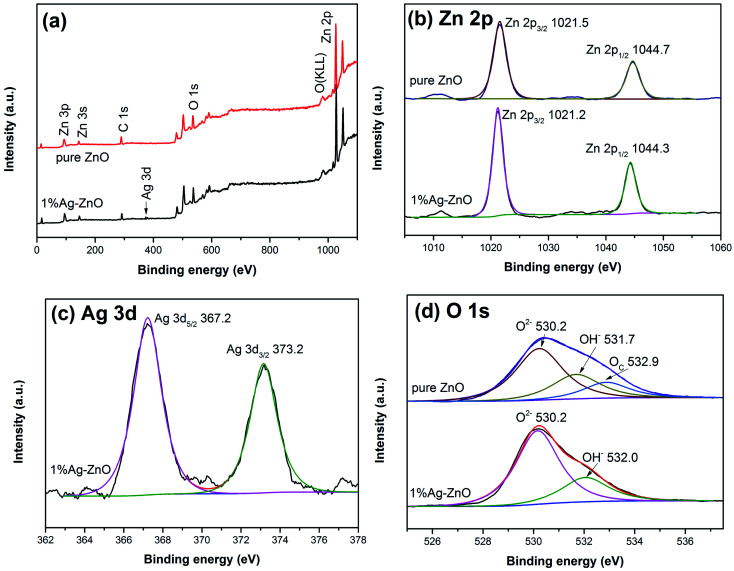
XPS spectra of pure ZnO and 1%Ag–ZnO (a) XPS full spectra of samples, (b) Zn 2p spectra, (c) Ag 3d spectrum, (d) O 1s spectra.

The full scan spectra of pure ZnO and 1%Ag–ZnO (Fig. 4(a)) show the signals from Zn, O, C and Zn, O, C, Ag elements, respectively. Among them, the presence of C element was probably due to the oil pollution from the instrument itself. Fig. 4(b) shows the high-resolution spectra of Zn 2p. The peaks of 1%Ag–ZnO are located at 1021.2 eV and 1044.3 eV, which are ascribed to Zn 2p_3/2_ and Zn 2p_1/2_, respectively, manifesting that Zn element exists in the Zn^2+^ chemical state in 1%Ag–ZnO.^24,30^ Both the peaks of Ag–ZnO shift to a lower binding energy compared to those of pure ZnO (1021.5 eV for Zn 2p_3/2_ and 1044.7 eV for Zn 2p_1/2_), which can be attributed to the electron transfer between ZnO and Ag.^31^ The Ag 3d spectrum of 1%Ag–ZnO (Fig. 4(c)) shows two peaks centered at 367.2 eV and 373.2 eV. The energy spacing between two peaks is 6.0 eV, indicating the presence of metallic Ag,^23,31^ which is consistent with the results of XRD analysis. The O 1s spectrum of pure ZnO (Fig. 4(d)) was decomposed into three peaks at 530.2 eV, 531.7 eV and 532.9 eV, which originate from the crystal lattice oxygen in ZnO, surface hydroxyl groups and chemisorbed oxygen,^32,33^ respectively. Furthermore, the O 1s spectrum of 1%Ag–ZnO is decomposed into two peaks at 530.2 eV and 532.0 eV, which are attributed to the lattice oxygen and surface hydroxyl groups, respectively.^1^ The surface hydroxyl groups content in a semiconductor increases after modification, which is favorable to the photocatalytic process.^34–36^ However, in our study, the amounts of surface hydroxyl groups of pure ZnO and 1%Ag–ZnO were calculated to be 27.0% and 26.3%, respectively, implying that Ag loading did not improve the content of surface hydroxyl groups.

### 3.4 Optical property

**Fig. 5:**
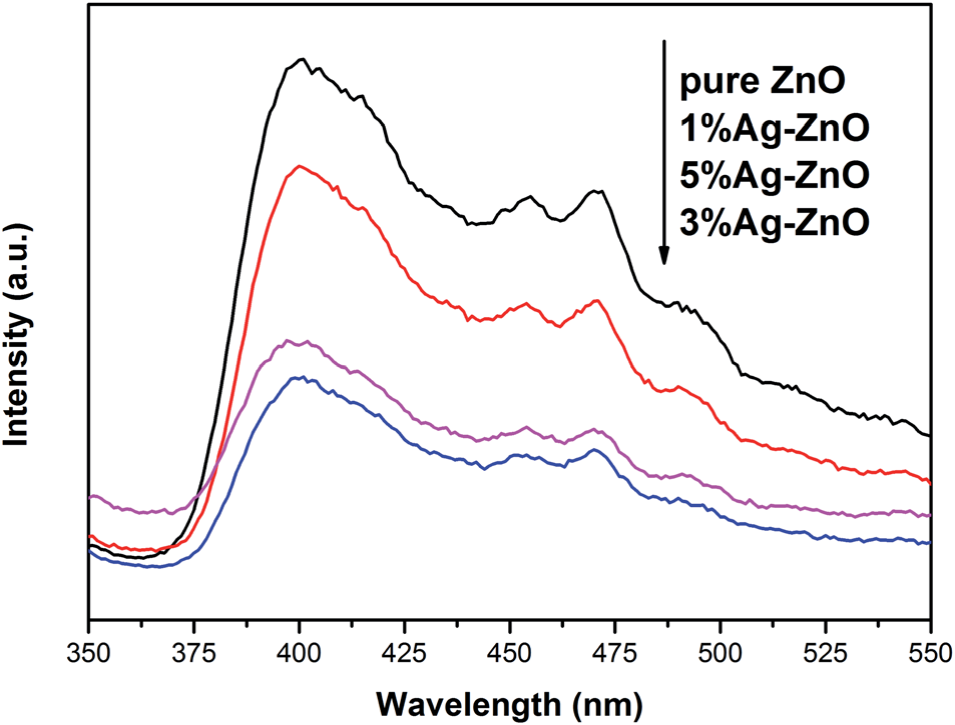
PL spectra of pure ZnO and Ag–ZnO.

Fig. 5 exhibits the PL spectra of pure ZnO and Ag–ZnO. The peak around 400 nm in pure ZnO can be ascribed to free excitonic emission.^9,37^ Several peaks at 450–500 nm are attributed to the transition between the different defect states.^9,24,37^ All the Ag–ZnO samples show lower peak intensity than pure ZnO, implying that the recombination rate of photoinduced pairs decreases *via* Ag modification, which is in favor of the photocatalytic activity improvement.^38^ The peak intensity of Ag–ZnO samples first decreases and then increases with the increase in the Ag content, which indicates that there is an optimum concentration for inhibiting the recombination of photoinduced electrons and holes. Some studies have shown that high doping concentration will form new recombination centers, which is not conducive to the separation of photoinduced electrons and holes.^9,19,28,30^ On the contrary, it has been reported that in a certain concentration range, the more doping, the more conducive to the separation of photoinduced pairs.^7,39^ In the present study, 3%Ag–ZnO exhibited the lowest peak intensity and excess Ag addition is unfavorable to charge separation.

**Fig. 6:**
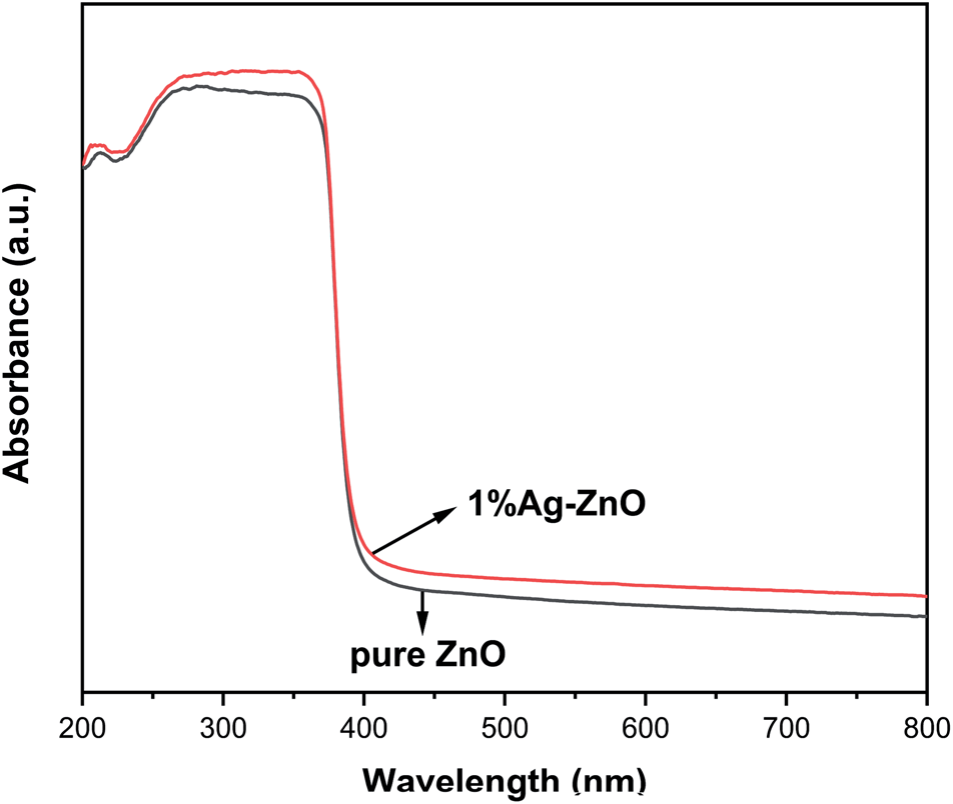
UV-visible absorption spectra of pure ZnO and 1%Ag–ZnO.

Fig. 6 exhibits the UV-vis absorption spectra of pure ZnO and 1%Ag–ZnO. Both the samples have strong absorption in the UV region. After Ag modification, the absorption edge of ZnO does not show a clear red-shift, indicating that Ag modification has little effect on the band gap width of ZnO. It is worth noting that the absorption of 1%Ag–ZnO in the visible region increases slightly, which is conducive to improving the photocatalytic performance under visible light.

### 3.5 Photocatalytic activity

**Fig. 7:**
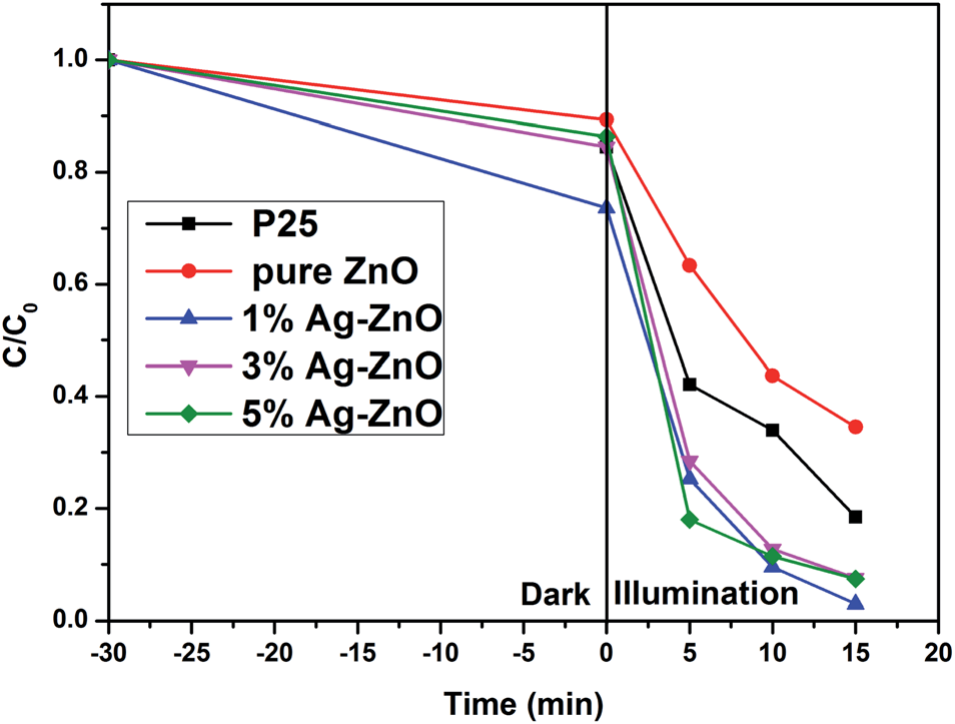
Photocatalytic degradation of MB by pure ZnO and Ag–ZnO.

The photocatalytic property of pure ZnO and Ag–ZnO nanocomposites was evaluated *via* the degradation of MB. Fig. 7 provides the degradation degrees of MB for pure ZnO and Ag–ZnO samples. Commercial P25 was employed for comparison, and its degradation degree was 81.5% after 15 min. The degradation degree of pure ZnO was 65.5%. Remarkably, Ag–ZnO photocatalysts showed a higher photocatalytic activity than commercial P25. The degradation degrees of 1%, 3% and 5%Ag–ZnO were 97.1%, 92.5% and 92.6%, respectively. 1%Ag–ZnO exhibited the highest photocatalytic activity, suggesting that an appropriate amount of Ag modification improves the photocatalytic activity significantly.

The surface area and surface hydroxyl group content of the photocatalyst are important to its photocatalytic activity. More surface area is beneficial for providing active reaction spots and enhancing light absorption. Surface hydroxyl group will generate superoxide radical ions (˙OH), which can decompose MB molecules effectively.^40^ However, in the present study, as discussed in BET and XPS sections, it was proven that the specific surface area and surface hydroxyl group content of ZnO remained almost unchanged after Ag loading. The increased photocatalytic activity should be attributed to the suppression of the photogenerated electrons and holes by Ag loading, which is confirmed by PL spectra. Besides, the increase in the visible light absorption is also the reason for the improvement of photocatalytic activity.

### 3.6 Photodegradation mechanism

**Fig. 8:**
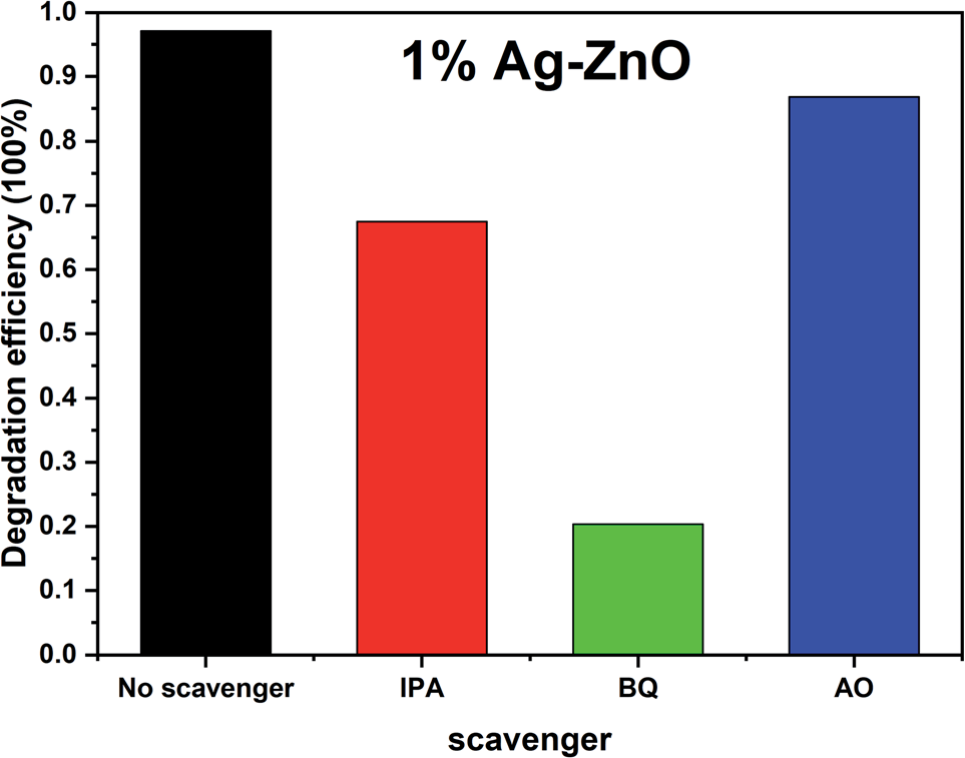
Effect of different scavengers on the degradation of RhB in 1%Ag–ZnO.

To investigate the active radicals in the photodegradation process, benzoquinone (BQ), isopropanol (IPA) and ammonium oxalate (AO) were added as scavengers to the photodegradation system, and the results are shown in Fig. 8. After adding BQ, IPA and AO, the degradation degrees of MB by 1%Ag–ZnO decreased to 20.3%, 67.4% and 86.8%, respectively. Since BQ, IPA and AO were selected to quench ˙O_2_^−^, ˙OH and h^+^, respectively, the results indicate that the ˙O_2_^−^ radical is the predominant active species in the photodegradation process. ˙OH and h^+^ species are the secondary factors.

**Fig. 9:**
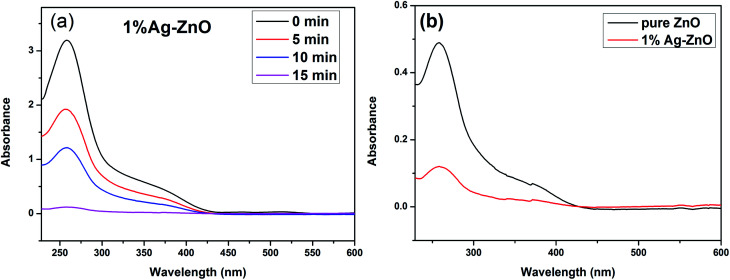
The NBT absorbance curves of 1%Ag–ZnO with increasing time (a), the comparison of pure ZnO and 1%Ag–ZnO (b).

As ˙O_2_^−^ radical is the main active species, therefore, the nitro blue tetrazolium (NBT) experiment was conducted to verify the formation of ˙O_2_^−^ species and compare the amount between pure ZnO and Ag–ZnO.^41,42^ Since NBT will react with ˙O_2_^−^ to form purple particles, which consumes NBT in the solution, resulting in a decrease in the NBT absorbance, therefore, the NBT absorbance gradually decreasing with an increase in the irradiation time (Fig. 9(a)) testifies the formation of ˙O_2_^−^ species in 1%Ag–ZnO under irradiation. Fig. 9(b) shows the NBT absorbance curves of pure ZnO and 1%Ag–ZnO at the same time. It is found that the NBT absorbance of 1%Ag–ZnO is lower than that of pure ZnO, which proves that more ˙O_2_^−^ is produced by 1%Ag–ZnO than pure ZnO. Ag modification is beneficial to the separation of photogenerated electrons and holes, which is consistent with the results from PL spectra.

**Fig. 10:**
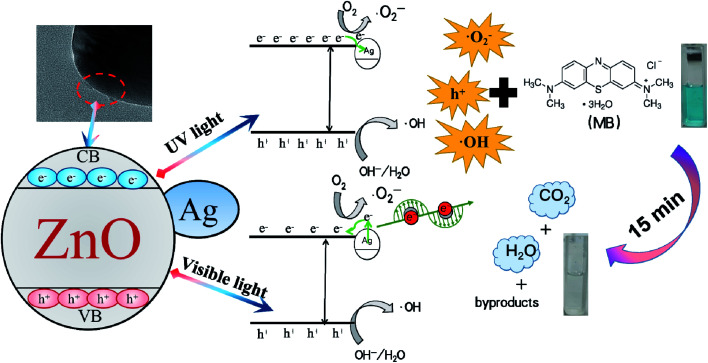
Photodegradation mechanism of 1%Ag–ZnO.

Based on the discussion above, a schematic for the photodegradation of RhB by 1%Ag–ZnO is proposed in Fig. 10. When ZnO is irradiated by a UV light source, electrons in the valence band (VB) will be excited to the conduction band (CB), leaving holes in VB simultaneously.^27,43^ The photogenerated electrons in CB will transfer to Ag particles as the CB energy level of ZnO is higher than the Fermi level of metallic Ag, which suppresses the recombination and prolongs the lifetime of photogenerated pairs.^28,30,40^ Under visible light, Ag particles are able to absorb visible light due to the effect of surface plasmon resonance and generate hot electrons. Part of them will jump to CB and react with O_2_ to generate ˙O_2_^−^ radicals.^44,45^ The electrons in the 3d orbit are easily transferred from Ag particles to CB of ZnO owing to the interface effect of Ag/ZnO heterojunctions,^46^ which will yield more ˙O_2_^−^ radicals and thus enhance the photocatalytic activity. In addition, the holes will transform into˙OH radicals. The ˙O_2_^−^, ˙OH radicals and holes will be responsible for decomposing the MB molecules effectively.^47–50^

Remarkably, the amount of Ag has an influence on the photocatalytic performance. The degradation degree declines slightly when the Ag/Ti molar ratio surpasses 1%. The decrease cannot be attributed to the formation of new recombination centers due to the addition of excess Ag because the PL peak intensity of 3%Ag–ZnO and 5%Ag–ZnO is still lower than that of 1%Ag–ZnO. It was confirmed that Ag particles are loaded on the ZnO surface and excess Ag particles will hinder the absorption of light,^51–53^ thus causing a drop in the photocatalytic activity. Table 1 lists the photocatalytic performance data reported in literature. The Ag–ZnO photocatalyst obtained in the present study possesses high photocatalytic activity.

**Table 1:** Photocatalytic efficiency of ZnO reported by references

Method	Catalyst	Target pollutant	Light source	Maximum efficiency	Ref.
One-step growth method	Pure ZnO, 400 mg L^−1^	MO (20 ppm)	High-pressure mercury lamp (360 W)	98.3% in 100 min	4
Chemical precipitation method	Ce–ZnO, 500 mg L^−1^	MO (5 mg L^−1^)	High-pressure mercury lamp (250 W)	89.5% in 240 min	7
Precipitation method	Pt–ZnO, 1000 mg L^−1^	RhB (20 mg L^−1^)	Ultra-Vitalux lamp (300 W)	96.9% in 60 min	13
Electrospinning method	Fe–ZnO, 400 mg L^−1^	MB (10 mg L^−1^)	Mercury lamp	88% in 360 min	15
Microwave assisted ultrasonicated precipitation method	Sn–Cu–ZnO, 1000 mg L^−1^	MB (20 ppm)	High-pressure halogen lamp (500 W)	98.5% in 180 min	16
Three-step hydrothermal and pulsed laser ablation method	Au–ZnO, 500 mg L^−1^	MB (5 × 10^−5^ M)	Xenon lamp (300 W)	98% in 20 min	19
Solvothermal method	Ag–ZnO, 8.3 mg L^−1^	MB (2.5 ppm)	High-pressure mercury vapor lamp (125 W)	99% in 120 min	20
One-pot hydrothermal method	Ag–ZnO, 200 mg L^−1^	RhB (1 × 10^−5^ M)	UV lamp (125 W)	92% in 80 min	24
Electrospinning and hydrothermal method	C–ZnO	RhB (10 mg L^−1^)	High-pressure mercury lamp (50 W)	96% in 50 min	33
Borohydride reduction method	Pd–ZnO, 500 mg L^−1^	Congo red (16 mg L^−1^)	UV light (100 W)	98.2% in 60 min	54
Sol–gel method	Ag–ZnO, 1000 mg L^−1^	MB (10 mg L^−1^)	Xenon lamp (250 W)	97.1% in 15 min	Present study

## 4 Conclusions

In the present study, pure ZnO and Ag–ZnO heterojunctions were synthesized *via* a sol–gel pathway and the photocatalytic performances were studied. The characterization results show that Ag^0^ nanoparticles deposit on the surface of ZnO and Ag/ZnO heterojunctions formed in the photocatalysts. Ag/ZnO photocatalysts show higher photocatalytic activity than commercial P25. Moreover, Ag loading suppresses the recombination of photoinduced electrons and holes effectively and enhances the visible light absorbance, thereby improving the photocatalytic activity. 1%Ag–ZnO exhibits the best photocatalytic performance, with the degradation degree of 97.1% after 15 min. Photocatalytic mechanism experiment results indicate that the main active species in the photodegradation process is the ˙O_2_^−^ radical.

## Conflicts of interest

The authors declare that they have no conflict of interest.

## Supplementary Material
